# Bronchoscopic intratumoral injection of tranexamic acid to prevent excessive bleeding during multiple forceps biopsies of lesions with a high risk of bleeding: a prospective case series

**DOI:** 10.1186/1471-2407-14-143

**Published:** 2014-03-01

**Authors:** Adil Zamani

**Affiliations:** 1 Department of Pulmonary Medicine, Meram Medical Faculty, Necmettin Erbakan University, Akyokus Mevkii, Meram 42080, Konya, Turkey

**Keywords:** Biopsy, Bronchoscopy, Hemorrhage, Lung cancer, Tranexamic acid

## Abstract

**Background:**

Significant bleeding may occur following endobronchial forceps biopsy or brushing of necrotic or hypervascular tumors in the airways. In some cases, methods such as endobronchial instillation of iced saline lavage and epinephrine may fail to control bleeding. The present study evaluated the efficacy and safety of a new bronchoscopic technique using intratumoral injection of tranexamic acid (IIT) for control of bleeding during forceps biopsy in patients with endobronchial tumors with a high risk of bleeding.

**Methods:**

The study was a prospective case series carried out in a single center. Bronchoscopic IIT was performed in those patients who had endoscopically visible tumoral lesions with persistent active bleeding following the first attempt at bronchoscopic sampling. Tranexamic acid (TEA) was injected through a 22-gauge Wang cytology needle into the lesion in nominal doses of 250–500 mg. After 2–3 minutes, multiple forceps biopsy specimens were obtained from the lesion.

**Results:**

Of the 57 consecutive patients included in the study, 20 patients (35.1%) underwent bronchoscopic IIT. The first attempt in 18 patients was endobronchial forceps biopsy (EBB), and because of a high risk of bleeding, the first attempt for the remaining two patients, who were on continuous dual antiplatelet therapy (aspirin and clopidogrel), employed endobronchial needle aspiration (EBNA) as a precautionary measure. Following IIT, subsequent specimens were obtained using EBB in all patients. Multiple forceps biopsy specimens (3–10) were obtained from the lesions (8 necrotic and 12 hypervascular) without incurring active bleeding. The following histopathologic diagnoses were made: squamous cell carcinoma (n = 14), adenocarcinoma (n = 2), small-cell lung cancer (n = 3), and malignant mesenchymal tumor (n = 1). No side effects of TEA were observed.

**Conclusions:**

Bronchoscopic IIT is a useful and safe technique for controlling significant bleeding from a forceps biopsy procedure and can be considered as a pre-biopsy injection for lesions with a high risk of bleeding.

**Trial registration:**

ISRCTN23323895

## Background

Optimal diagnostic yield in patients with endoscopically visible tumors requires multiple forceps biopsies (at least five specimens) [1]. However, some centrally located endobronchial tumors (necrotic or hypervascular) may bleed significantly after the first biopsy attempt. In our clinic, we generally use endobronchial instillation of iced saline and epinephrine in patients with persistent biopsy-related bleeding, but in some cases this method may fail to achieve endobronchial hemostasis.

A new bronchoscopic technique, the use of intratumoral injection of tranexamic acid (IIT), has recently been described. The effectiveness of bronchoscopic IIT at controlling significant bleeding during forceps biopsy procedures was demonstrated in two cases with centrally located necrotic endobronchial tumors [2]. The aim of this prospective case series was to assess further the efficacy and safety of this technique in a larger number of patients with endobronchial tumors. IIT is not part of standard care at our institution and was therefore performed for the purpose of this research.

## Methods

The study was designed as a prospective case series study and carried out at a single center.

### Study population

Patients (≥18 years) suspected to have lung cancer because of signs, symptoms, chest radiograph, and computed tomography and/or fluorodeoxyglucose positron-emission tomography findings underwent fiberoptic bronchoscopy. Prior to the procedure, patients were screened to assess liver and kidney functions; blood counts; and coagulation studies, including platelet count, prothrombin time, and activated partial thromboplastin time.

Patients who had any of the following conditions were excluded from the study: unfitness to undergo an endoscopy, unwillingness to provide written informed consent, history or risk of thrombosis, active thromboembolic disease, subarachnoid hemorrhage, a known or suspected bleeding disorder, platelet count < 150,000/mm^3^, international normalized ratio > 1.3, renal failure (blood urea nitrogen > 30 mg/dL and/or serum creatinine > 2.0 mg/dL), history or physical evidence of liver disease, and disturbances of color vision.

The study protocol for off-label use of TEA was approved by Meram Medical Faculty Ethical Committee (approval number 2009/327), and each patient provided fully informed, written consent before enrollment.

### Bronchoscopy

After premedication with atropine 0.5 mg subcutaneously and conscious sedation with midazolam 2–4 mg intravenously, fiberoptic bronchoscopy using an Olympus BF-1T60 bronchoscope (Olympus Corp., Tokyo, Japan) was performed transnasally under local anesthesia (2% lidocaine) with the patients in sitting position. Supplemental oxygen was delivered at 2–5 L/min via nasal cannula. Oxygen saturation and vital signs were monitored continuously in all patients throughout the procedure. Biopsy specimens were obtained with disposable oval-cup forceps (jaw outside diameter 1.8 mm).

### Bronchoscopic IIT

Bronchoscopic IIT was performed in those patients with endoscopically visible tumoral lesions (necrotic or hypervascular) who had had persistent endobronchial bleeding following the first attempt at bronchoscopic sampling. Persistent endobronchial bleeding was defined as requiring continuous suctioning for ≥ 2 min [3]. In 18 patients, the first attempt was performed using endobronchial forceps biopsy (EBB) technique. The remaining two patients had drug-eluting coronary stents and were on continuous dual-antiplatelet therapy (aspirin and clopidogrel), and upon consultation with the cardiology department, these drugs were not recommended to be stopped before bronchoscopy. Therefore, in these patients, because of the high risk of bleeding, the first attempt was performed using endobronchial needle aspiration (EBNA) as a precautionary measure. Following IIT, subsequent specimens were obtained by EBB in all patients. It is often difficult to accurately measure blood loss because of the bleeding into the bronchial tree. Therefore, in the present study, a quantitative measurement of the volume of bleeding was not provided.

Tranexamic acid (TEA) (Transamine ampoule 250 mg/2.5 mL; Fako Drugs Ltd., Turkey) was injected through a 22-gauge × 13-mm Wang cytology needle (MW-122, ConMed, Billerica, MA, USA) into the lesion in fractional amounts at various points (two to seven insertions) in nominal doses of 250 to 500 mg (because the total internal volume of the MW-122 was found to be ~1.3 mL , the actual amount of TEA delivered to tumor tissue ranged between approximately 120 and 370 mg). After 2–3 minutes, multiple forceps biopsy specimens were obtained from the lesion. All patients were followed up for a week after bronchoscopic procedures.

## Results

Between October 2009 and July 2012, 57 consecutive patients were included in this study. Of these, 20 patients (35.1%; mean age ± standard deviation [SD], 61.6 ± 10 years; range, 41–80 years) met inclusion criteria and underwent bronchoscopic IIT. Tumors were located predominantly in the right bronchial system (65%). Airway obstruction varied between 50% and 100%. Multiple forceps biopsy specimens (mean number ± SD, 5.7 ± 1.8; range, 3–10) were obtained from the tumoral lesions (8 necrotic and 12 hypervascular) of all patients without producing active bleeding. No procedure-related complications or side effects of TEA were observed. The following histopathologic diagnoses were made: squamous-cell carcinoma (n = 14), adenocarcinoma (n = 2), small-cell lung cancer (n = 3), and malignant mesenchymal tumor (n = 1). Non-small-cell lung cancer constituted 80% of all lung cancers (Table 1). No adverse effects of TEA were reported during the follow-up period.

**Table 1:** Data of the patients who underwent bronchoscopic IIT

**Case #, gender, age(years)**	**Location of lesion**	**AO (approx. %)**	**TEA nd/dd (mg)**	**EBB (n)**	**Histology**
** Necrotic tumor (n = 8)**					
1 M(73)	RMB	80	250/120	10	SqCC
2 M(41)	RMB	90	250/120	5	SqCC
3 M(66)	RB3	100	250/120	3	SqCC
4 M(64)	RLLB	100	500/370	6	SqCC
5 M(72)	RBB	100	500/370	3	SqCC
6 M(58)	RULB	100	500/370	6	SqCC
7 M(64)	RULB	90	500/370	5	AC
8 M(80)*	LMB	100	500/370	5	MMT
** Hypervascular tumor (n = 12)**					
9 M(79)	RBI	70	250/120	4	SqCC
10 M(58)*	RMLB	90	250/120	3	SCLC
11 M(53)	LULB	100	500/370	4	SqCC
12 M(61)	LB4	80	500/370	5	SCLC
13 M(47)	RMB	90	500/370	6	SqCC
14 M(50)	LULB	50	500/370	7	AC
15 M(62)	RMB	90	500/370	9	SqCC
16 M(60)	Trachea-LMB	50-80	500/370	6	SqCC
17 M(59)	LULB	80	500/370	6	SCLC
18 M(64)	RULB	100	500/370	6	SqCC
19 M(53)	LULB	50	500/370	7	SqCC
20 M(67)	RBI	60	500/370	7	SqCC

*Continuous dual antiplatelet therapy (aspirin and clopidogrel).

AC- adenocarcinoma; AO-airway obstruction; dd-approximate delivered dose; EBB-endobronchial forceps biopsy; LB4- superior lingular segmental bronchus; LMB- left main bronchus; LULB- left upper lobe bronchus; MMT- malignant mesenchymal tumor; nd-nominal dose; RBB-right basal bronchus; RBI- Right bronchus intermedius; RB3- right upper lobe anterior segmental bronchus; RLLB -right lower lobe bronchus; RMB - right main bronchus; RMLB-right middle lobe bronchus; RULB-right upper lobe bronchus; SCLC- small-cell lung carcinoma; SqCC- squamous cell carcinoma; TEA-tranexamic acid.

## Discussion

The present study demonstrates that bronchoscopic IIT is effective for control of significant bleeding during multiple forceps biopsies of endobronchial necrotic or hypervascular bronchogenic tumors.

Fiberoptic bronchoscopy is generally a safe and well-tolerated procedure. However, there is increased risk when a bronchoscopic biopsy is performed [4]. The complication of bronchoscopy-related bleeding commonly occurs and is the most challenging for a bronchoscopist to manage. Reported rates of bronchoscopy-induced bleeding vary widely, ranging from less than 1% to approximately 20% [5].

Malignant lesions of the airways are more likely than benign mucosal lesions to bleed upon biopsy. In particular, necrotic or various hypervascular tumors tend to bleed significantly during forceps biopsy or brush biopsy [6]. In some cases, blood loss following endobronchial biopsy may be greater than 200 mL [7].

For control of persistent biopsy-related bleeding, the British Thoracic Society recommends topical instillation of small amounts of 1:10,000 epinephrine solution [1]. However, because of potential systemic absorption and adverse events (tachyarrhythmia, vasoconstriction, hypertension), the amount of epinephrine should be limited [5]. Moreover, when administered into the lung periphery, epinephrine can even lead to a potentially fatal arrhythmia [8]. Terlipressin, a derivative of vasopressin, is another vasoconstrictor that has been used in control of bronchoscopy-related bleeding. However, endobronchial application may also have cardiovascular effects, such as increase in heart rate and decreased mean arterial pressure [3].

Recently, endobronchial instillation of TEA has been shown to be highly effective in treating massive bleeding (600–750 mL) following bronchoscopic procedures (transbronchial biopsy and electrocautery) in two patients with malignant tumors. The bleeding stopped immediately after bolus endobronchial instillation of TEA (500–1,000 mg) [9]. In another study, hemostasis was achieved in 14 cancer patients with iatrogenic bleeding after endobronchial instillation of 15 mL of saline solution containing 500 mg of TEA [10].

The present study differs from the above-mentioned studies in two respects. First, the successful control of biopsy-induced bleeding was achieved by a different route of administration of TEA in all patients. Although the actual amount of TEA (120–370 mg) delivered to tumor tissue was less than in the above-mentioned studies, intratumoral concentration would be expected to be much higher than extratumoral concentration. Moreover, multiple deep injections in tumoral tissue provided better distribution of TEA and allowed multiple biopsy samples to be taken without active bleeding. Second, in the present study two patients were on continuous dual-antiplatelet therapy (aspirin and clopidogrel). It has been reported that clopidogrel with or without aspirin significantly increases the risk of bleeding after transbronchial lung biopsy, and its discontinuation before the procedure has been recommended [11]. Interestingly, in our two patients in whom dual-antiplatelet therapy was not discontinued, multiple forceps biopsies were performed without causing active bleeding; however, this finding should be confirmed in future studies with a larger number of patients.

The diagnostic yield from EBB for an exophytic mass lesion varies between 67% and 100%. Several factors can decrease the yield, such as surface necrosis of the tumor, sampling error, inadequate tissue, or presence of crush artifact [12]. Particularly for necrotic-appearing tumors, some authors recommend the addition of EBNA to EBB to obtain a specimen from the core of the lesion [13]. One study reported the diagnostic yield of EBB and combination EBB + EBNA for exophytic mass lesions to be 72% and 84%, respectively. According to that study, no difference in diagnostic yield was observed between EBNA performed before and after EBB [12]. Therefore, taking samples by EBNA in addition to those taken by EBB following IIT may be considered to increase diagnostic yield.

TEA, a synthetic derivative of the amino acid lysine, exerts antifibrinolytic activity by reversibly binding to plasminogen and blocking its interaction with fibrin, thereby preventing dissolution of the fibrin clot. It reduces perioperative blood loss and transfusion requirements in a variety of clinical settings, including cardiac surgery, major orthopedic surgery, and gynecological conditions, and decreases mortality rates in trauma patients with significant bleeding [14,15]. In addition, current best evidence indicates that TEA may reduce both the duration and volume of bleeding in patients with hemoptysis [16]. Generally, in clinical practice, suggested dosages of TEA are 10 mg/kg intravenously given three to four times daily for 2–8 days or orally as two 650-mg tablets three times a day for a maximum of 5 days [17]. TEA is taken up by various tissues, and the highest concentrations have been found in the lungs, kidneys, and liver [14,18]. An antifibrinolytic concentration remains in different tissues for as long as 17 hours [19]. Adverse events associated with TEA are uncommon; gastrointestinal effects including nausea and diarrhea have been reported with oral administration, and hypotension has occasionally been reported with rapid intravenous administration [20,21]. In the present study, bronchoscopic IIT was well tolerated by all patients without occurrence of adverse events. It is also noteworthy that TEA is very inexpensive (Table 2).

**Table 2:** Comparison of intratumoral injection of TEA and some hemostatic agents used in bronchoscopic procedure related bleeding

	TEA*	**Epinephrine **[5]** (1:1,000)**	**Terlipressin **[3]
Dosage	250-500 mg	2 ml	1 mg
Untoward reactions	None	Vasoconstriction	Increase in heart rate
		Hypertension Tachyarrhythmias	Decrease in blood pressure
Other possible properties	Antitumor [22]		
	Partial reversion of platelet aggregation dysfunction due to antiplatelet therapy with aspirin and clopidogrel [23]		
Cost ($) [24]	1.25-2.50	1.84	29.86

*TEA**-**tranexamic acid.

In the present study, the dosing of TEA was determined according to previously published studies [9,10] in which the investigators used 500–1,000 mg of the drug with successful outcomes. Because of the internal volume of the MW-122, the actual amount of TEA (120–370 mg) delivered to tumor tissue was less than the smallest amounts administered in the above-mentioned studies. Nevertheless, biopsy-induced bleeding was successfully control with this dose range in all our patients.

In the era of personalized medicine, acquisition of sufficient quality and quantity of tumor tissue for histologic diagnosis and molecular testing (epidermal growth factor receptor mutations and anaplastic lymphoma kinase fusions) is becoming increasingly important for the treatment of patients with non-small cell lung cancer [25,26]. However, although obtaining a greater number of biopsy samples leads to greater diagnostic accuracy, it also leads to a greater risk of bleeding [25]. Bronchoscopic IIT may help facilitate the procurement of adequate tissue from endobronchial bronchogenic tumors that have a high risk of bleeding. This technique, possibly, may also be effective for metastatic tumors with significant risk of bleeding, such as renal cell carcinoma or carcinoid tumors and others with a particular tendency toward hypervascularity. Although some authors suggest the use of vasoactive agents (e.g., epinephrine) to minimize bleeding during biopsy of a carcinoid tumor [6], endobronchial administration of epinephrine should be avoided because it may worsen the catecholamine response and precipitate coronary spasm [27].

## Conclusions

In conclusion, bronchoscopic IIT is a safe technique for controlling significant bleeding after a forceps biopsy procedure and may be considered as a pre-biopsy injection for endobronchial necrotic or hypervascular bronchogenic tumors. Following IIT, multiple biopsies can be performed without significant bleeding. This increases patient comfort and safety and reduces the duration of a procedure. Further prospective studies are needed to assess the effectiveness of IIT for bronchoscopy patients with bleeding risk factors (e.g., continuous dual-antiplatelet therapy or coagulation disorders).

## Competing interests

Dr. Adil Zamani has no conflict of interest or financial ties to disclose.

## Pre-publication history

The pre-publication history for this paper can be accessed here:

http://www.biomedcentral.com/1471-2407/14/143/prepub
